# MAR4 *Streptomyces*: A Unique Resource for Natural Product Discovery

**DOI:** 10.1021/acs.jnatprod.3c01007

**Published:** 2024-02-14

**Authors:** Douglas Sweeney, Alexander B. Chase, Alexander Bogdanov, Paul R. Jensen

**Affiliations:** † Scripps Institution of Oceanography, University of California, San Diego, La Jolla, California 92093, United States; ‡ Department of Earth Sciences, Southern Methodist University, Dallas, Texas 75275, United States

## Abstract

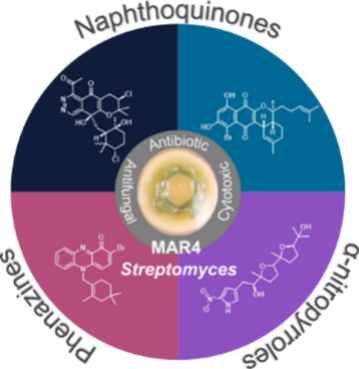

Marine-derived *Streptomyces* have long been recognized as a source of novel, pharmaceutically relevant natural products. Among these bacteria, the MAR4 clade within the genus *Streptomyces* has been identified as metabolically rich, yielding over 93 different compounds to date. MAR4 strains are particularly noteworthy for the production of halogenated hybrid isoprenoid natural products, a relatively rare class of bacterial metabolites that possess a wide range of biological activities. MAR4 genomes are enriched in vanadium haloperoxidase and prenyltransferase genes, thus accounting for the production of these compounds. Functional characterization of the enzymes encoded in MAR4 genomes has advanced our understanding of halogenated, hybrid isoprenoid biosynthesis. Despite the exceptional biosynthetic capabilities of MAR4 bacteria, the large body of research they have stimulated has yet to be compiled. Here we review 35 years of natural product research on MAR4 strains and update the molecular diversity of this unique group of bacteria.

## Introduction

The genus *Streptomyces* (phylum: Actinobacteria) is a highly diverse lineage of Gram-positive bacteria commonly isolated from soils and is estimated to have emerged along with flowering plants ca. 380 million years ago (mya).^1^ Early investigations were largely driven by the remarkable capacity of these bacteria to produce biologically active natural products, leading to important discoveries such as the antibiotic streptomycin,^2^ the chemotherapeutic drug doxorubicin,^3^ and the antiparasitic agent ivermectin,^4^ among myriad others. By 1970, over 3000 *Streptomyces* species had been described,^5^ while a 2015 revision reduced this number to 553.^6^*Streptomyces* species richness is reflected in their genomic diversity, with strains across the genus sharing as little as 76% average nucleotide identity (ANI), a value comparable to some bacterial families.^7,8^ Consequently, it has been suggested that the genus should be split into six genera,^9^ the largest of which would still remain the most speciose bacterial genus described to date.^9^

Mirroring their species diversity, *Streptomyces* are associated with diverse habitats and ecosystem functions including the recycling of recalcitrant organic matter in soils.^10−12^ While they are also known as symbionts^13^ and pathogens,^14^ they can be readily isolated from marine habitats,^15,16^ where little is known about their ecology. Evidence that *Streptomyces* are metabolically active in marine habitats is largely lacking and confounded by their production of resistant spores, which may remain dormant when introduced into seawater.^17^ Yet, *Streptomyces* salt tolerance is well documented, with 98% of 1300 non-marine strains growing at typical seawater salinities,^18^ suggesting that even strains introduced into marine systems could be metabolically active. While distinguishing among transient and obligate marine *Streptomyces* remains challenging, one study revealed that 30% of the isolates from coastal areas required seawater for growth, providing evidence that some strains are adapted to the marine environment.^19^ While more work is required to understand how *Streptomyces* adapt to marine biomes, these adaptations may differ from those observed in Gram-negative marine bacteria, as has been shown for the obligate marine actinomycete genus *Salinispora*.^20^ Despite these unknowns, the common recovery of *Streptomyces* from marine samples and their ability to grow at seawater salinities suggest that many can be metabolically active in marine systems and that some have likely evolved marine adaptations that affect specialized metabolism.

This concept is best reflected in an unusual group of largely marine-derived *Streptomyces* identified in the 1980s^21,22^ and termed “MAR4”.^23^ In contrast to their terrestrial counterparts, MAR4 strains are enriched in the production of halogenated hybrid isoprenoid (HI) natural products including prenylated naphthoquinones, phenazines, and α-nitropyrroles.^23^ These compounds are of particular interest, as the introduction of isoprene groups can increase affinity for biological membranes, thus potentiating the bioactivity of the “core” molecule.^24^ In addition, halogens can act as a leaving group,^25^ thus facilitating interactions with nucleophiles and further potentiating the biological activity of HI natural products. The unique capacity for HI production among MAR4 strains is supported by the large numbers of prenyltransferase (PTase) enzymes encoded in their genomes.^26^ More specifically, the analysis of 13 MAR4 genomes revealed that all strains possessed PTase genes and that they averaged five per genome.^26^ In contrast, only 29% of non-MAR4 *Streptomyces* genomes encoded a single PTase, and those that had them averaged one per strain.

**Chart 1:**
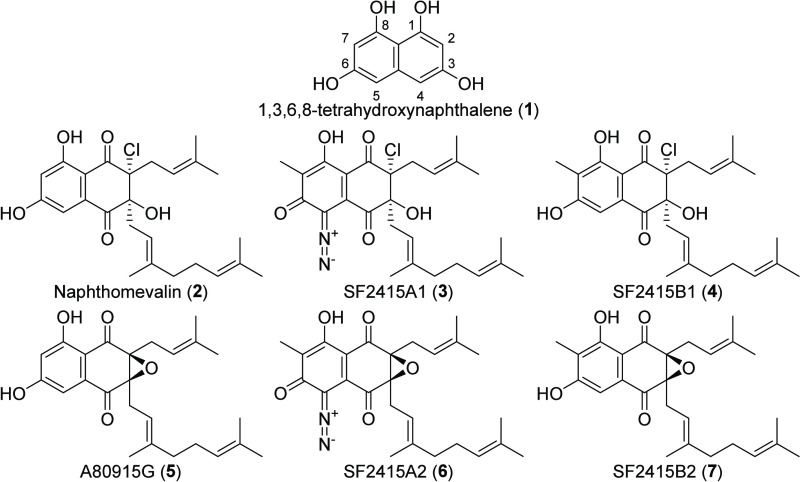
Possible Intermediates in Napyradiomycin Biosynthesisa

a Dihydronaphthoquinones (**2**–**7**) have been reported from MAR4 strains, while THN (**1**) has not.

**Chart 2:**
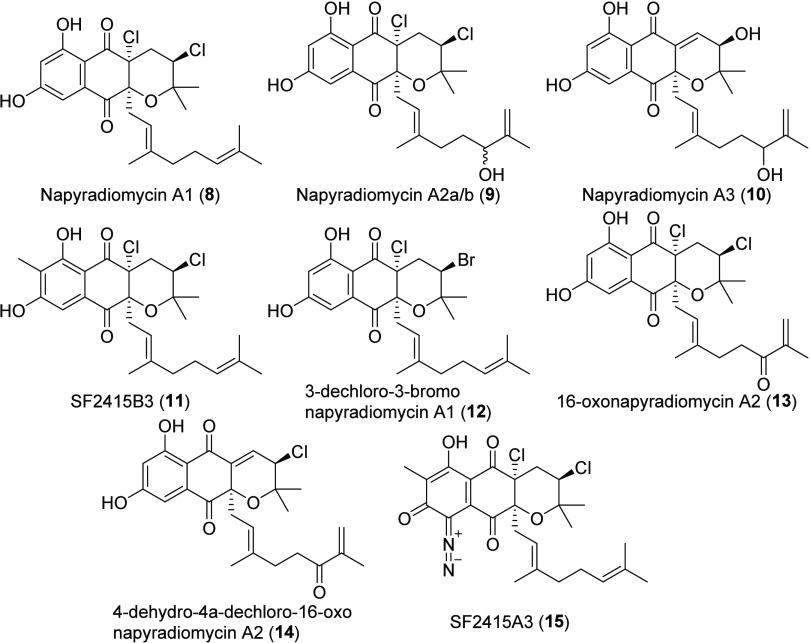
MAR4 A-Type Napyradiomycins

**Chart 3:**
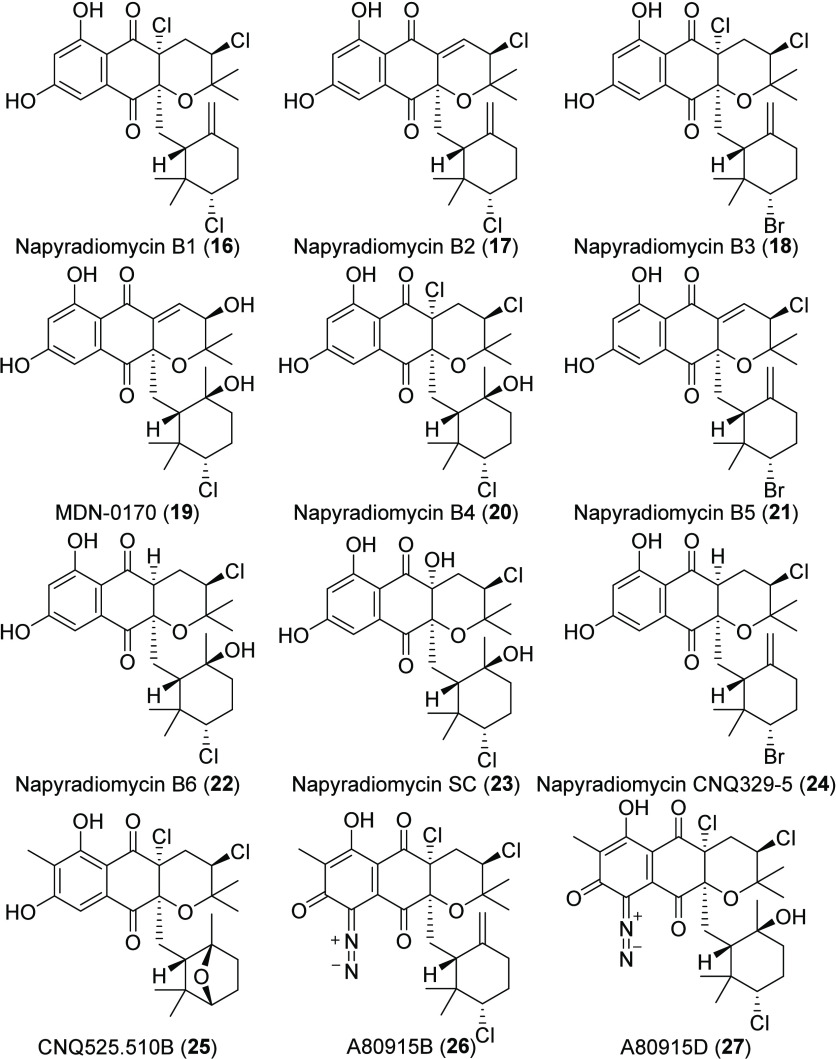
MAR4 B-Type Napyradiomycins

**Chart 4:**
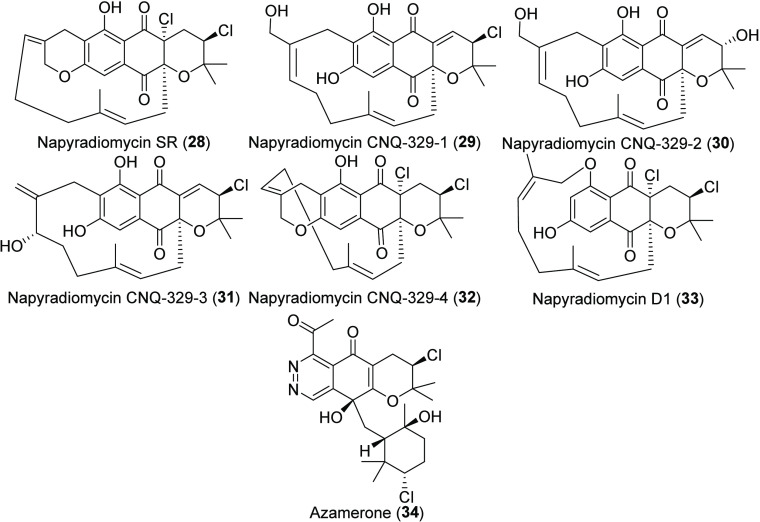
MAR4 C-Type and D-Type Napyradiomycins and Azamerone

**Chart 5:**
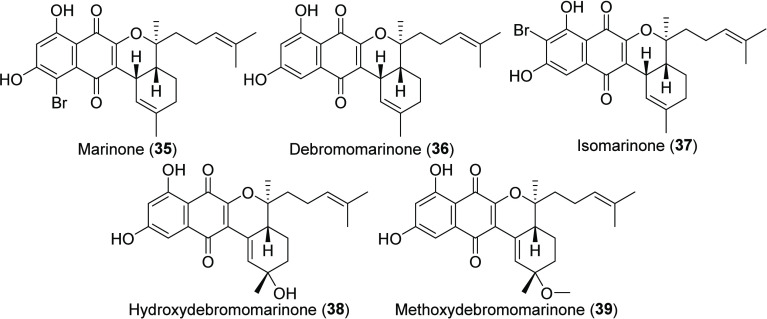
Marinone Class of THN Natural Products

**Chart 6:**
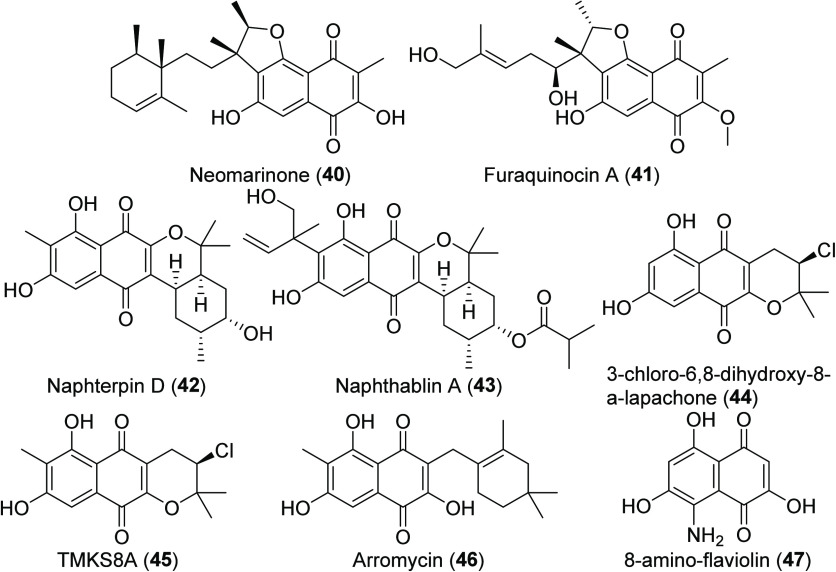
Unclassified THN Products Reported from MAR4 Strainsa

a Furaquinocin A (**41**) is shown for reference.

**Chart 7:**
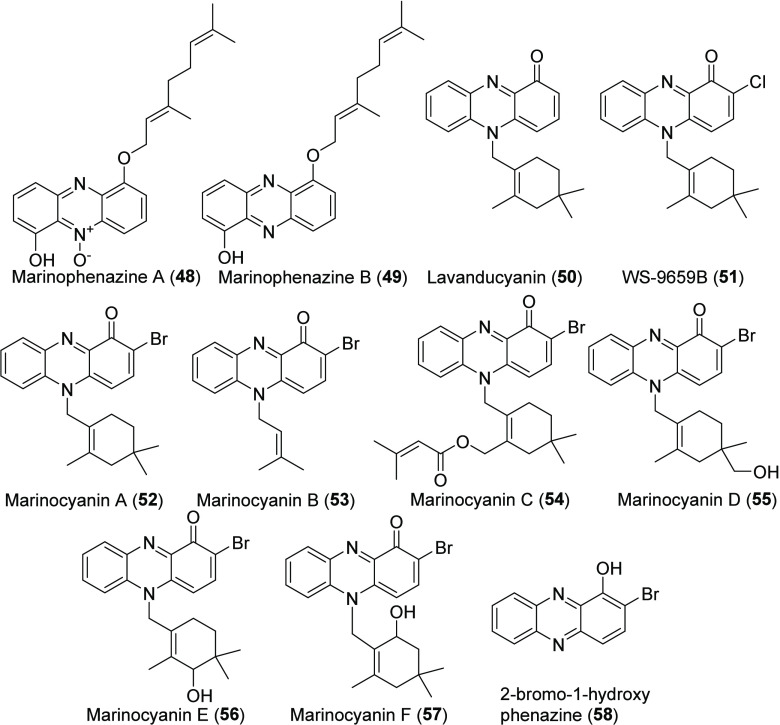
MAR4 Phenazine Natural Products

**Chart 8:**
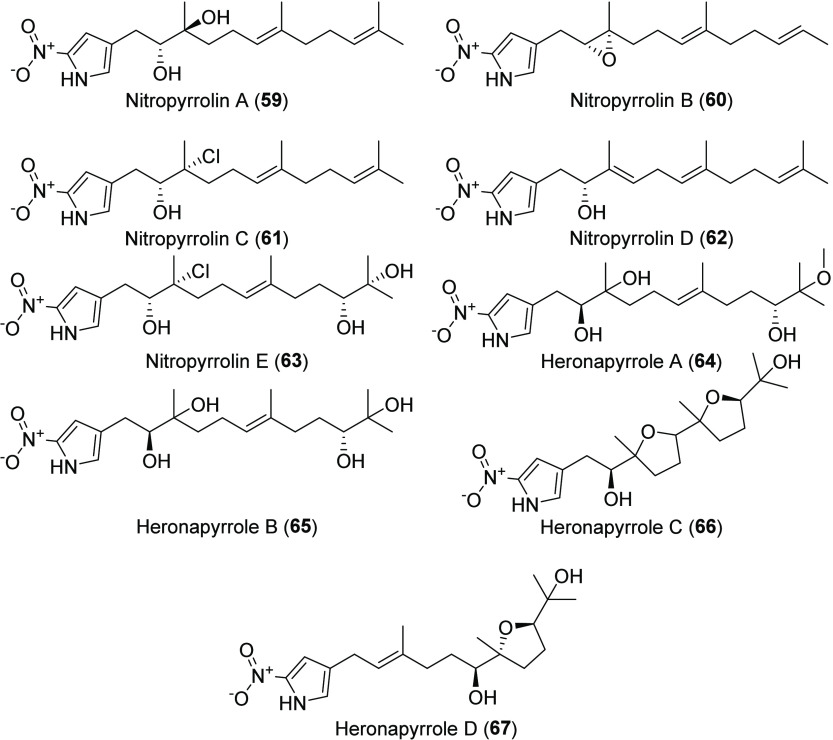
MAR4 α-Nitropyrrole Natural Products

MAR4 strains have also helped to expand our mechanistic understanding of natural product biosynthesis. For example, the first prokaryotic vanadium-dependent haloperoxidase (VHPO) enzyme was identified from a MAR4 strain.^27^ These enzymes were previously known only from red algae, where they generate positively charged bromonium and chloronium ions that facilitate halogen incorporation into natural products.^27^ Unlike the algal enzymes, MAR4 VHPOs incorporate halogens in regio- and stereospecific manners that are difficult or impossible to replicate synthetically, making them of great interest for biocatalysis.^28^

The only prior analysis of MAR4 diversity assessed both cultured and culture-independent reports.^29^ The culture-independent assessment used targeted PCR primers, with the results suggesting that considerable diversity remained uncultured.^29^ The MAR4 16S rRNA clade established in this study included the *S. aculeolatus* and *S. synnematoformans* type strains. These are the only named species in the clade, and they demarcate the two major subclades in the phylogeny. The *S. aculeolatus* type strain was isolated in 1987 from a soil sample collected in coastal Japan.^21^ Similar to other *Streptomyces*, this isolate tolerated 3% sodium chloride,^21^ suggesting the potential for growth in the marine environment. *S. aculeolatus* strains produce a variety of napyradiomycin analogs and possible intermediates with various antibiotic activities against Gram-positive bacteria.^21,30^ The *S. synnematoformans* type strain was isolated in 2007 from a sand dune near the shores of the brackish Lake Mariout in Egypt and was reported to grow in 7% sodium chloride.^31^ This species produces compounds in the marinone class,^22,30^ which are structurally related to the napyradiomycins, thus suggesting a shared evolutionary history.

In this meta-analysis, we provide an updated assessment of the diversity and distribution of bacteria in the MAR4 clade, the compounds they produce, their associated biosynthetic gene clusters (BGCs) when known, and the biosynthetic studies they have inspired. By mining public data sets, we find that the MAR4 clade is largely marine, more diverse than previously recognized, and occurs from temperate to tropical locations across the globe. These bacteria have been an exceptional source of halogenated HI natural products, with the potential for many more discoveries to come.

## MAR4 Diversity and Distribution

### MAR4 Diversity

**Figure 1:**
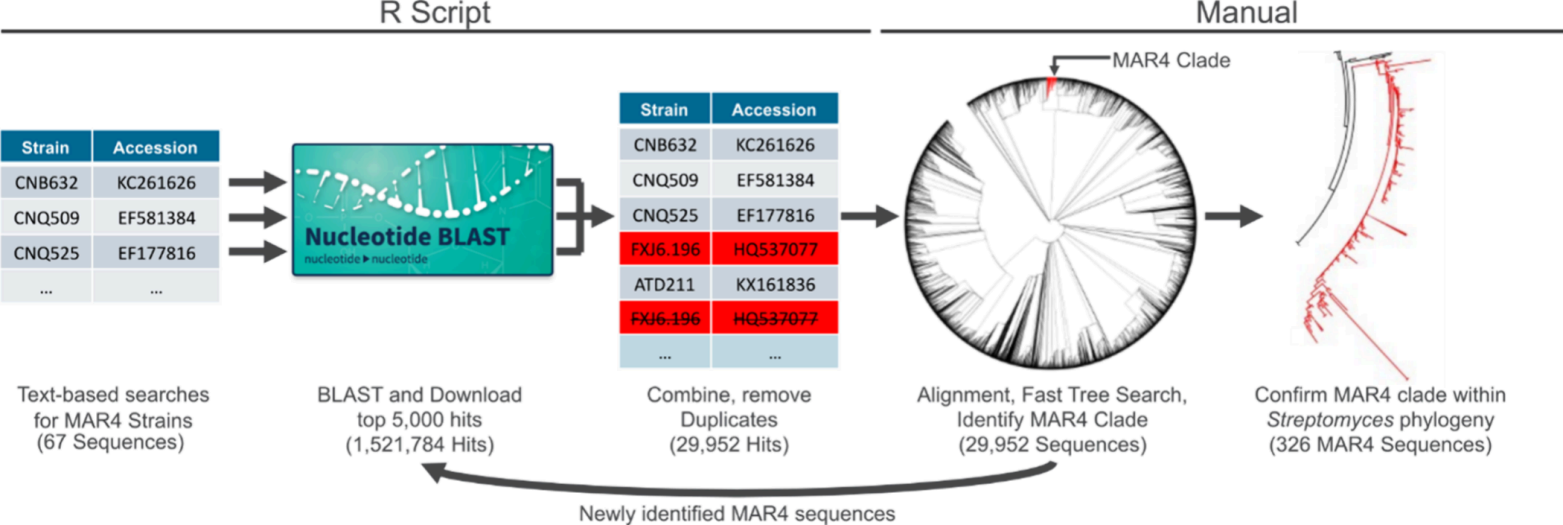
Workflow used to identify MAR4 16S rRNA sequences. The results from iterative, NCBI database searches were dereplicated and used to generate preliminary phylogenies. Candidate MAR4 sequences were used as queries to repeat the search process until no new sequences were identified. The final phylogeny (Figure S1) revealed a monophyletic clade consisting of 326 MAR4 sequences.

**Figure 2:**
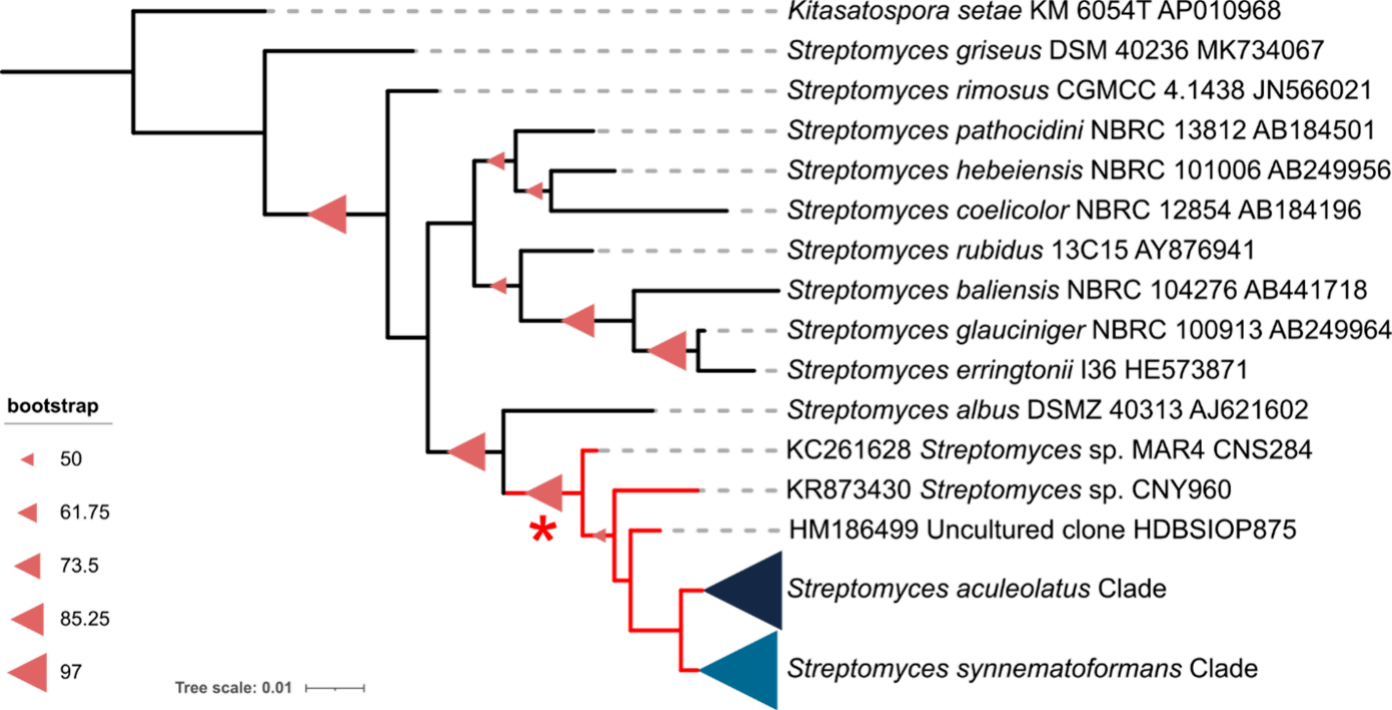
MAR4 16S phylogeny generated using centroid sequences (98% clustering). Red lines demarcate the MAR4 clade. The *S. aculeolatus* and *S. synnematoformans* clades, which account for 167 and 14 of the sequences, respectively, have been collapsed. The 10 most closely related *Streptomyces* species are included for reference. Bootstrap values are shown as red triangles. * indicates the basal node that defines the MAR4 clade.

Six rounds of recursive NCBI BLAST searches generated 1,521,784 candidate MAR4 16S rRNA gene sequences that were dereplicated to 29,952 unique sequences (Figure 1). These results were combined with 721 sequences obtained from BLAST searches of the JGI-IMG database using the 16S rRNA sequences of the *Streptomyces aculeolatus* SF2415 and *S. synnematoformans* S155 type strains. Phylogenetic analyses placed 326 of these sequences in the MAR4 clade and expand the size of the clade by 38% from prior estimates (Table S1). These sequences correspond to 127 cultured strains (some strains have more than one 16S sequence) and 171 clones (uncultured). Included in this total are 11 strains for which genome sequences are available but were not found using the BLAST-based approach due to incomplete 16S rRNA sequences. The 16S rRNA sequences within the clade are diverse, sharing as little as 93.1% sequence similarity. Within the 16S rRNA phylogeny, the two type strains demarcate two highly populated but poorly supported clades within the lineage (Figures 2, S1). To increase support for these clades, the sequences were clustered at 98% and a phylogeny was generated from the centroids. Within this phylogeny, most of the new MAR4 sequences fall within the *S. aculeolatus* clade.

### Geographic Distributions

**Figure 3:**
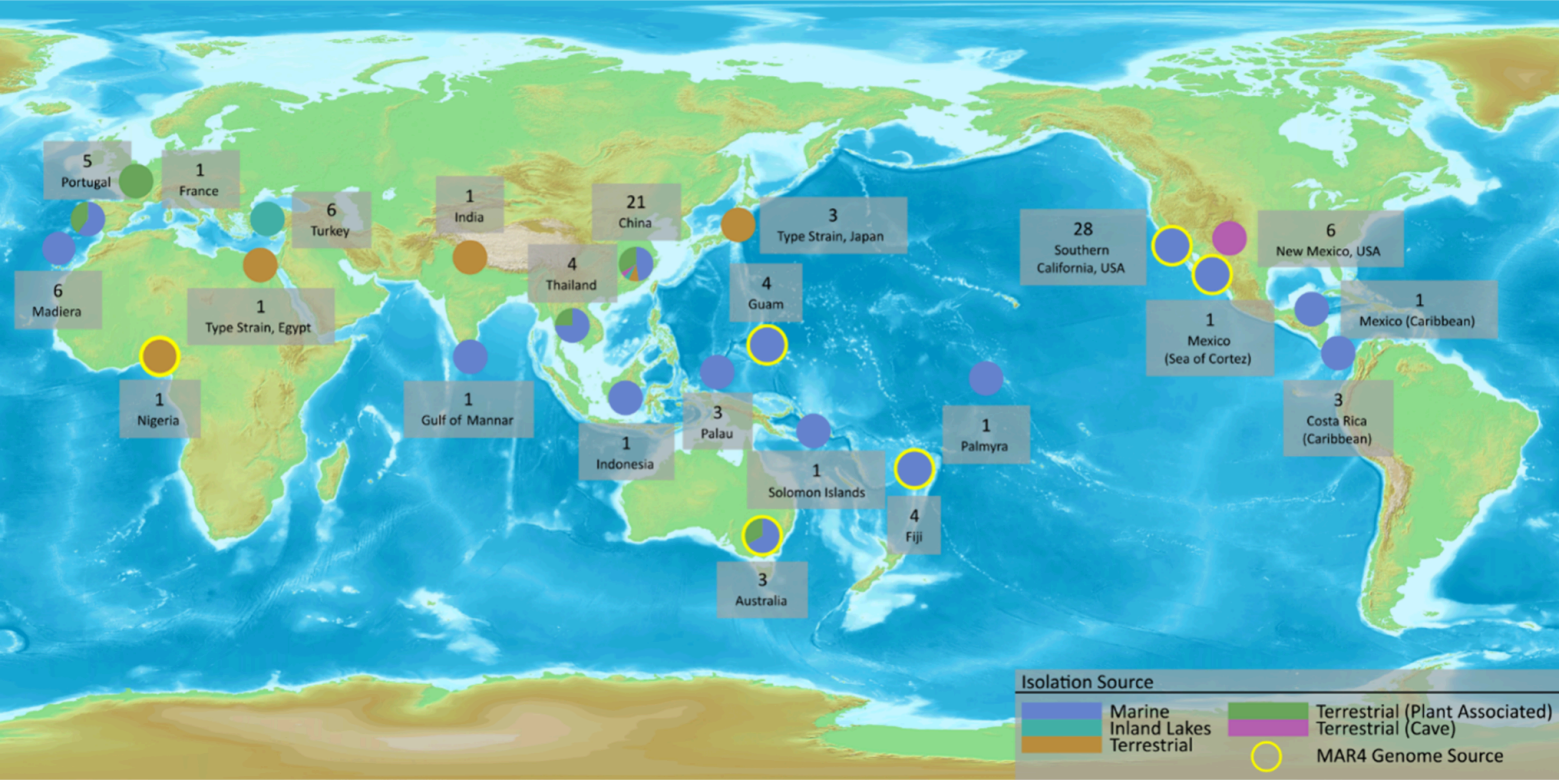
Global distribution and source of MAR4 strains. Pie charts represent isolation source from each location; yellow borders indicate strains with genome sequences. Location and number of strains are indicated.

While the geographic distributions of the MAR4 sequences are influenced by sampling biases, it is nonetheless notable that 83% (*N* = 272) originate from marine habitats (Figure 3). Most of these sequences (*N* = 168) were clones derived from a study that revealed extensive and yet to be cultured MAR4 diversity in marine sediments.^29^ We focused the remainder of this study on cultured MAR4 strains, given our aim of reviewing the natural products reported from this group. Among these, 84 of 127 had sufficient metadata to determine whether they were of marine origin. Most of these were recovered from marine sediments including sediments collected at depths up to 1900 m (Table S1). Many were isolated from estuarine sediments, where it becomes especially difficult to distinguish between terrestrial and marine bacteria. In addition to sediments, MAR4 strains have also been reported from sponges and ascidians.^32^ Among the 106 MAR4 strains with sufficient metadata to determine their geographic origin, most were isolated from the Pacific Ocean. Yet they have also been recovered from the Caribbean off Costa Rica,^33^ La Bocana, Mexico, off the coast of Portugal (Estremadura), the Madeira archipelago,^34^ and the Gulf of Mannar in the Indian Ocean. It remains unclear whether the lack of MAR4 strains from more polar regions represents limits to their distributions or sampling biases.

Among the 36 MAR4 strains derived from terrestrial samples, six were sourced from saline to hypersaline Turkish endorheic crater lake sediments (Table S1). Others were isolated from coastal environments and, thus, are not easily categorized. Twelve were described as rhizospheric or endophytic across a range of plant hosts, which may represent another MAR4 ecological niche. Six strains were reported as endemic to the Lechuguilla Cave, a large underground system that remained isolated from the surface until its discovery in 1986.^35,36^ The presence of fossilized algae, brachiopods, and other marine organisms within the cave hints at an active marine ecosystem that disappeared roughly 260 mya, which corresponds to the estimated divergence time of the MAR4 clade within the *Streptomyces*.^36^ These strains could provide evolutionary insights into the early differentiation of the MAR4 lineage should genome sequences become available. Interestingly, all *S. synnematoformans* strains were isolated from samples collected from marine sources or close to the sea; however, some *S. aculeolatus* strains have been reported from inland soils in China, India, Nigeria, and Turkey, perhaps indicating that this lineage is better adapted for non-saline growth.^37^

## Natural Products Reported from MAR4 Strains

Natural products reported from the MAR4 clade were compiled by cross-referencing strain identifiers with the published literature. In total, 22 of the 127 MAR4 strains could be linked to 93 compounds representing six molecular families (Chart S1, Table S2): tetrahydroxynaphthalene (THN)-based naphthoquinones, phenazines, α-nitropyrroles, furanones, polyethers, and chromenones. Notably, 84 (90%) of these compounds were prenylated, and 61 (66%) were halogenated, demonstrating the exceptional capacity of these bacteria to produce halogenated, hybrid isoprenoid natural products. Below, we present the molecules reported from MAR4 strains in the context of these six molecular families, with an emphasis on notable bioactivities and biosynthetic discoveries.

Most compounds reported from MAR4 strains (70%) are derived from a 1,3,6,8-tetrahydroxynaphthalene (THN) (**1**) precursor (Chart 1). This precursor is produced by a type III polyketide synthase (T3PKS) as opposed to the type I polyketide synthase (T1PKS) employed in fungal THN natural products.^38^ These uniquely derivatized molecules include the naphthomevalins,^39^ napyradiomycins,^40−46^ marinones,^22,45,47^ and a group of compounds we have called “unclassified THN products” that include naphthablins,^48^ naphterpins,^49^ and neomarinone.^47,50^ Prior reports suggested that MAR4 THN natural products are produced in a clade-specific manner, with the marinones and napyradiomycins produced by the *S. synnematoformans* and *S. aculeolatus* clades, respectively.^29^ However, as more strains have been analyzed, these patterns have not been supported.^34^ It is likely that our understanding of how natural products and their associated BGCs are distributed through the MAR4 clade will continue to evolve as more data are acquired.

### Napyradiomycins

Napyradiomycins are prenylated naphthoquinones originally reported in 1986 as antibacterial metabolites from a non-MAR4 strain of *Streptomyces rubra* (formerly *Chania rubra*).^51^ Subsequently, they have become the largest family of THN-based meroterpenoids with over 50 analogs reported.^52^ Much of the napyradiomycin structural diversity is derived from the cyclization patterns of the prenyl and geranyl side chains, which is used to delineate napyradiomycins into types A–D as described below. These four types do not include likely biosynthetic intermediates such as naphthomevalin (**2**) and analogs (**3**–**7**) (Chart 1, Table S2), which possess linear C-2 prenyl and C-3 geranyl groups and likely represent the simplest napyradiomycins reported. Additional diversity arises from the degree of oxygenation, the number and position of halogens, and C-7 methylation of the THN core. MAR4 strains produce all four napyradiomycin types (A–D) along with these apparent biosynthetic intermediates.^52^

A-Type napyradiomycins (Chart 2, Table S2) are tricyclic molecules defined by cyclized C-2 prenyl and linear C-3 geranyl substituents attached to the THN core, as typified by napyradiomycin A1 (**8**). Thirteen A-type napyradiomycins have been isolated from MAR4 strains. They differ in the number, type, and position of halogenation, prenylation, oxidation, and diazotization (**8**–**15**, Chart 2, Table S2). While the installation of halogens in the A-type napyradiomycins has been linked to VHPOs, it is unclear whether the absence of halogens (or their replacement with hydroxy groups as in napyradiomycin A3 (**10**)) results from spontaneous loss events or alternative biosynthetic pathways. Additional diversity is generated from the presence or absence of C-7 methylation of the THN core as in SF2415B3 (**11**) and SF2415A3 (**15**) and five of the 12 A-type napyradiomycins. The A-type napyradiomycins lacking these methyl groups contain a hydrogen at this position, as seen in napyradiomycin A1 (**8**). SF2415A3 (**15**) is the only MAR4 A-type napyradiomycin that contains a diazo moiety, while non-MAR4 strains have been shown to produce other A-type napyradiomycins with this functional group (e.g., 7-demethyl SF2415A3 from *S. antimycoticus* NT17^53^).

B-Type napyradiomycins (Chart 3) are tetra- and pentacyclic molecules with cyclized C-2 prenyl and C-3 geranyl substituents attached to the THN core, as typified by napyradiomycin B1 (**16**). The B-type napyradiomycins are the most numerous, with 25 of the >30 derivatives reported from MAR4 strains. Much of the diversity within this group is introduced via nonspecific oxidation of the terpene moiety following halonium ion induced cyclization of the geranyl subunit.^27^ Further diversity is observed in the halogenation patterns of the analogs (**17**–**27**). As in the A-type napyradiomycins, it is unclear if the absence of halogens in the tricyclic core of some compounds (**10**, **19**) represents spontaneous loss or an enzyme-mediated process. This will likely remain unclear until more genetic information becomes available. Fifteen of the MAR4 B-type napyradiomycins including A80915D (**27**) display methylation at C-7, which presumably prevents conversion to the C- or D-types. B-Type napyradiomycins A80915B (**26**) and A80915D (**27**) reported from MAR4 strains are the only described B-type napyradiomycins to contain a diazo moiety.

C-Type napyradiomycins (Chart 4) are tetra- and pentacyclic molecules in which one of the terminal methyl groups of the geranyl substituent is cyclized with C-7 of the THN core, as in napyradiomycin SR (**28**). To date, five C-type napyradiomycins have been reported from MAR4 strains (Chart 4, Table S1). Oxidation of the geranyl moiety and halogenation in the tricyclic core generate added structural diversity within this group (**28**–**32**); however, the enzymes responsible have yet to be identified. Interestingly, in napyradiomycin SR (**28**) and napyradiomycin CNQ-329-4 (**32**), the geranyl moiety is further cyclized with the hydroxy group at C-6 of the THN core to create pentacyclic compounds. No C-type napyradiomycins have been reported to contain a diazo moiety. Napyradiomycin D1 (**33**) is the only D-type napyradiomycin reported to date (Chart 4), and it was discovered from a *Streptomyces* strain we have identified as belonging to the MAR4 clade.^54^ This molecule displays an unusual cyclization between the geranyl moiety and the hydroxy group at C-8 of the THN core. Following precedent, we have grouped the unusual phthalazinone-containing molecule azamerone (**34**) with the napyradiomycins,^40,52,55^ as it has been proposed that this molecule is produced by the napyradiomycin BGC.^28,55^ This tetracyclic molecule differs from other napyradiomycins in that it has a phthalazinone core and a migration of the terpene subunit to C-4 of the THN core.

Napyradiomycins possess potent antibiotic activity against many Gram-positive and some Gram-negative bacteria (Table S2). They also have low micromolar activity against various cancer cell lines (Table S2). Notably the A- and B-type napyradiomycins, along with those containing a diazo group, consistently show the greatest activity; however, the mechanism for these enhanced activities remains unknown (Table S2). Interestingly, azamerone is less active than other diazo-containing napyradiomycins, possibly because the diazo group is embedded within a six-membered ring (Table S2).

The hybrid polyketide-isoprenoid origin of the napyradiomycins was demonstrated in 1987 using ^13^C-labeling.^56^ In 2006, it was established that the THN component of a related hybrid isoprenoid compound originates from a T3PKS, while the isoprene units originate from the mevalonate pathway,^57^ suggesting that the same biosynthetic basis may apply to the napyradiomycins. Using THN synthase and prenyltransferase genes as biosynthetic hooks, the first napyradiomycin BGCs were discovered in MAR4 strains CNQ-525 and *S. aculeolatus* NRRL 18422.^27^ Both of these BGCs supported a T3PKS origin for the THN core as well as the mevalonate pathway as the source of the terpene units in the MAR4 napyradiomycins. Surprisingly, this BGC included three VHPO genes, which until this time had only been observed in fungi and marine algae.^27^ The encoded enzymes catalyze the halonium-induced cyclization of the geranyl moieties in B-type napyradiomycins. Further studies established that both prenylation steps occur before the VHPO-catalyzed cyclization reactions, supporting the suggestion that the naphthomevalins and A-type napyradiomycins represent biosynthetic intermediates for which the terpene units are cyclized to produce the B-, C-, and D-type napyradiomycins.^58^ Perhaps the most biosynthetically interesting napyradiomycins are those containing a diazo functionality attached to the THN core.^52,55,59^ While these molecules have been proposed as azamerone (**34**) intermediates,^40^ the late stages of azamerone biosynthesis, as well as the installation of the diazo group in some napyradiomycins, are not fully understood.^40,55^

### Marinones

The marinones (Chart 5) represent a second class of THN natural products reported from MAR4 strains.^22^ While structurally similar to the napyradiomycins, marinone (**35**) and its non-halogenated congener debromomarinone (**36**) differ from the napyradiomycins by the presence of a farnesyl-derived and cyclized C-3 side chain and the possible migration of hydroxy groups on the THN core. Both marinone and debromomarinone possess low μg/mL antibiotic activity and cytotoxicity (Table S2). Three additional marinones (**37**–**39**) were subsequently discovered along with neomarinone (**40**, Chart 6), which was originally reported to possess a new carbon skeleton, but the structure was subsequently revised.^47,50^ Given that the positions of the prenyl substituents in neomarinone (**40**) are most closely aligned with those of the furaquinocins (**41**) (Chart 6), we included it with the “unclassified THN products”. Marinones have only been reported from the *S. synnematoformans* clade within the MAR4 group, although structurally similar molecules such as the naphthterpins and naphthablins (Chart 6) have been reported from both MAR4 and non-MAR4 *Streptomyces* alike.^49,52^ The naphthterpins (**42**) and naphthablins (**43**) differ from the marinones by the incorporation of shorter geranyl as opposed to farnesyl terpene units, and we have grouped them along with neomarinone with the “unclassified THN products”.

It is interesting that marinone biosynthesis remains unresolved despite the structural similarities (THN precursor and prenylation) to the napyradiomycins. Surprisingly, ^13^C labeling studies demonstrated that the prenyl groups in the marinones are derived from the non-mevalonate pathway in contrast to the mevalonate pathway reported in napyradiomycin biosynthesis.^50^ More strains will need to be studied to determine whether these patterns are consistent. Further biosynthetic differences between the napyradiomycins and the marinones, naphterpins, and naphthablins include a presumed C-3 to C-2 oxygen migration in the flaviolin precursor of the marinone, naphterpin, and naphthablin molecular families.^58^ This reaction could possibly be achieved through an epoxide intermediate, as observed in some naphthomevalin analogs (Chart 1), although the mechanism by which the C-2 oxygen is installed remains to be determined. While the marinone BGC has yet to be identified, a candidate encoding a THN synthase, three VHPOs, and the non-mevalonate pathway for terpene biosynthesis was identified in a debromomarinone-producing MAR4 strain.^60^ In vitro characterization of the VHPO enzymes in this BGC revealed that the reactions catalyzed mimicked the proposed marinone biosynthetic pathway and suggested a role for cryptic halogenation in the biosynthesis of non-halogenated marinone end products.^58^

### Unclassified THN Products

Additional “unclassified THN products” produced by MAR4 strains (Chart 6) include the marinone-like naphterpins (**42**) and naphthablins (**43**) as well as napyradiomycin-like molecules such as 3-chloro-6,8-dihydroxy-8-α-lapachone (**44**) and TMKS8A (**45**). Other molecules such as arromycin (**46**) and 8-amino-flaviolin (**47**) remain difficult to classify, and only 8-amino-flaviolin has been associated with a BGC. In the case of 8-amino-flaviolin (**47**), an intermediate in napyradiomycin biosynthesis,^61^ the amino group at C-5 of the THN core suggests it may also be an intermediate in the production of the MAR4 A- and B-type diazo compounds (**15**, **26**, **27**). While these diazo compounds are believed to be azamerone precursors,^28,55^ the linkage between 8-amino-flaviolin, diazo napyradiomycins and azamerone remains to be experimentally validated. Neomarinone (**40**) has previously been included in the marinone class; however, neomarinone lacks the oxygen migration seen in the marinone, naphterpins, and naphthablins. It is also structurally unique among the MAR4 THN hybrid isoprenoids in that the isoprene unit is connected to C-6 of the flaviolin core, as in the non-MAR4 compound furaquinocin (**41**). Also unlike the marinones, neomarinone (**40**) has been reported from strains outside of the MAR4 clade, providing further support for its inclusion with “unclassified THN products.”^29,45^ Arromycin (**46**) presents an interesting anomaly in that it possesses a cyclolavandulyl moiety attached to C-2 of the THN core. Finally, the α-lapachones (**44**, **45**) isolated from MAR4 strains CA-271078, SCSIO 10428, and TMKS8 may represent shunt products from napyradiomycin biosynthesis, where premature cyclization of the C-2 isoprene unit could prevent attachment of a C-3 geranyl substituent obnserved in napyradiomycins. However, more work is needed to understand how their biosynthesis is related to the napyradiomycins and marinones.

### Phenazine Natural Products: Marinophenazines and Lavanducyanins

The first phenazine natural products reported from a MAR4 strain, and perhaps the most unusual, are marinophenazines A and B (**48**, **49**) (Chart 7).^62^ These compounds provide rare examples of HIs in which a geranyl moiety is attached to the O-6 position of the phenazine core.^62,63^ Marinophenazine B and its *O*-methyl derivative were subsequently reported from the marine-derived *S. niveus* strain SCSIO 3406, which is outside of the MAR4 clade.^64^ These compounds were named phenaziterpenes A and B, respectively, the latter of which has not been reported from a MAR4 strain.

MAR4 strains also produce phenazines in the lavanducyanin family (Chart 7). Lavanducyanin (**50**) was first reported from the non-MAR4 *Streptomyces* strain CL190 isolated from Ishigaki Island, Japan, in 1989^65^ and subsequently from other *Streptomyces*.^53,66^ That same year, lavanducyanin (named WS-9659A) along with its chlorinated derivative WS-9659B (**51**) were reported from an unidentified *Streptomyces* strain.^67^ In 2017, MAR4 strains isolated from marine sediments were reported to produce **50**, **51**, and the novel, brominated lavanducyanins marinocyanin A–F (**52**–**57**).^68^ To date, brominated compounds in the lavanducyanin family have only been reported from MAR4 strains.

Phenazine natural products have been implicated in electron transport, biofilm formation, and the regulation of virulence genes.^69^ The prenylated phenazine lavanducyanin inhibits testosterone-5α reductase, an enzyme targeted in the treatment of prostate hyperplasia.^67^ Further testing showed that halogenated lavanducyanin analogs had antibacterial, antifungal, and cytotoxic activity, with molecules containing both prenyl and halogen moieties showing the greatest activity^68^ (Table S2). Interestingly, subtoxic doses of lavanducyanin (**50**) stimulate HeLa and murine cell proliferation^70^ (Table S2), thus demonstrating the importance of exploring dose–activity relationships.

In 2014, Zeyhle et al. identified a candidate marinophenazine BGC in the CNQ-509 genome and provided *in vitro* evidence that the membrane-bound *O*-prenyltransferase CnqPT1 catalyzes the biosynthesis of marinophenazines from 1,6-dihydrophenazine and geranyl diphosphate.^63^ Curiously, the phenazine biosynthesis genes and the PTase were not colocalized but instead found in two separate gene clusters.^63^ Unlike the marinophenazines, the marinocyanins and lavanducyanin have yet to be linked to a BGC. However, biosynthetic studies have shown that the cyclolavandulyl terpene moiety of lavanducyanin is formed before attachment to the phenazine skeleton, unlike similarly cyclized terpene moieties in the napyradiomycin class that are cyclized after attachment.^71^ Phenazines such as 2-bromo-1-hydroxyphenazine (**58**) detected in MAR4 strains^68^ likely represent non-prenylated intermediates in the production of the marinocyanins.

### α-Nitropyrroles

Nitropyrrolins A–E (Chart 8, **59**–**63**) isolated from MAR4 strain CNQ-509 represent the first terpenyl-α-nitropyrrole natural products described.^72^ They display varying levels of oxidation and halogenation on the farnesyl side chain. Subsequently, Raju et al. isolated the related heronapyrroles A–C (**64**–**67**) from a MAR4 strain (CMB-StM0423) cultured from a marine sediment sample collected near Heron Island, Australia.^73^ Heronapyrrole C (**66**) is unique among the nitropyrrolins in that the farnesyl moiety is cyclized to form two tetrahydrofuran rings.^73^ Heronapyrrole production was stimulated by a diketopiperazine produced by a fungal contaminant, suggesting it may act as a defense against fungal competitors.^74^ While nitropyrrolin D showed modest HCT-116 cytotoxicity,^72^ no cytotoxic activity was reported for the heronapyrroles.^73^ A candidate heronapyrrole BGC that has homology to a BGC in the nitropyrrolin-producing strain CNQ-509 was identified in the CMB-StM0423 genome; however, experimental verification linking these genes to nitropyrrolin or heronapyrrole biosynthesis has yet to be obtained.^75^

### Actinofuranones

**Chart 9:**
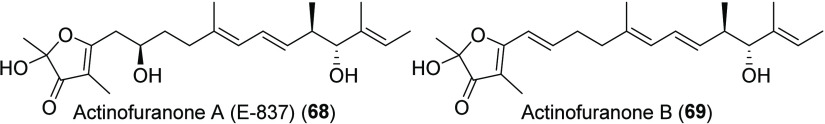
MAR4 Actinofuranone Natural Products

Actinofuranones A and B (**68**, **69**, Chart 9) were reported from a marine sediment derived MAR4 strain.^76^ These polyketides contain a 3-furanone ring with a C-5 alkyl side chain and are structurally related to siphonareienfuranone, which was isolated from a marine invertebrate, and the aurafurons, which were isolated from terrestrial myxobacteria.^76^ Shortly after the publication of actinofuranones A and B, a molecule with the same structure as actinofuranone A was described from *S. aculeolatus* NRRL18422 and named E-837.^77^ In that study, the proprietary *Streptomyces* strain Eco86 produced two related furanones, E-492 and E-975.^77^ Due to the lack of public sequence data for this strain, it cannot be determined if it belongs to the MAR4 clade. Actinofuranones C and D-I were subsequently reported from the non-MAR4 actinomycetes *Amycolatopsis* #AC43^78^ and *S. gramineus*.^79^ While furanones have been implicated in quorum sensing,^80^ a biological role for the actinofuranones has not been established. The “E” compound showed moderate electron transport chain inhibition in eukaryotes.^77^ Anti-inflammatory activity was demonstrated for other members of this class via the inhibition of NO production in RAW 264.7 macrophage cells.^79^ The actinofuranone BGC was identified in the genomes of the “E” compound producers *S. aculeolatus* NRRL18422 and *Streptomyces* Eco86 based on bioinformatics to include a nine-module T1PKS. Notably, both of these BGCs contained an unusual flavin monooxygenase implicated in furanone biosynthesis via an unusual cyclic carbonate intermediate.^77^

### Tetronomycin

**Chart 10:**
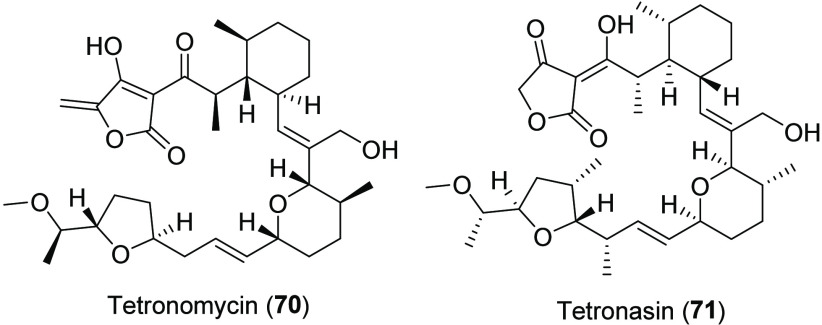
Structures of Tetronomycin and Tetronasin

The production of tetronomycin (**70**) (Chart 10) was initially mentioned in the description of napyradiomycin derivatives produced by the MAR4 strain *S. aculeolatus* A80915.^59^ This polyether ionophore was initially isolated in 1981 from the non-MAR4 strain *Streptomyces* sp. NRRL11266^81^ and shown to transport metal cations across plasma membranes and be broadly active against Gram-positive bacteria.^81^ Interestingly, tetronomycin (**70**) shares a similar planar structure with tetronasin (**71**) from *S. longisporoflavus*, but with the opposite configuration at every equivalent stereocenter.^82^ Tetronasin strongly binds to sodium ions and is used in the cattle industry to promote weight gain.^83,84^ The tetronasin-producing strain *Streptomyces* sp. CP26-58^85^ was found to clade with the MAR4 strains in our 16S rRNA phylogeny, showing for the first time that MAR4 strains are capable of producing both tetronomycin and tetronasin. The tetronomycin type-I modular PKS BGC was identified from cosmid libraries using a ketosynthase-specific DNA hybridization probe.^82^

### Chromenones

**Chart 11:**
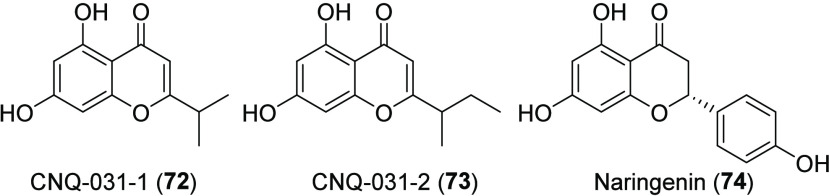
Chromenone Natural Products from MAR4 Strain CNQ-031a

The most recent additions to the compendium of MAR4 natural products are two chromenone-derived molecules (**72**, **73**, Chart 11) produced by strain CNQ-031, which was isolated from marine sediments collected off the coast of California.^86^ These molecules possess reversible monoamine oxidase inhibition (MAOI) activity, with CNQ-031-1 (**72**) being the most potent and selective inhibitor (Table S2). While the BGC has yet to be identified, these molecules are structurally similar to the T3PKS-derived molecule naringenin (**74**), a well-known plant metabolite, and previously reported from a non-MAR4 strain of *S. clavuligerus*.^87^ The biosynthesis of the MAR4 chromenones could be explained using a similar biosynthetic pathway with modified leucine and isoleucine starter units instead of a modified tyrosine.

a Naringenin is a related chromenone produced by plants, fungi, and other *Streptomyces* strains.

## Analysis of Public Genomes

Using the 18 MAR4 genome sequences identified in our analyses, a multigene phylogeny was generated and ANI values were calculated to assess species-level diversity within the clade. We identified six clades that shared <95% ANI, a common threshold used for species delineation^88^ (Figure S2). This suggests that, in addition to *S. aculeolatus* and *S. synnematoformans* (Figure S2), four additional MAR4 species await formal description. Given the limited number of genomes currently available, we suspect that additional MAR4 species diversity remains uncharacterized.

**Figure 4:**
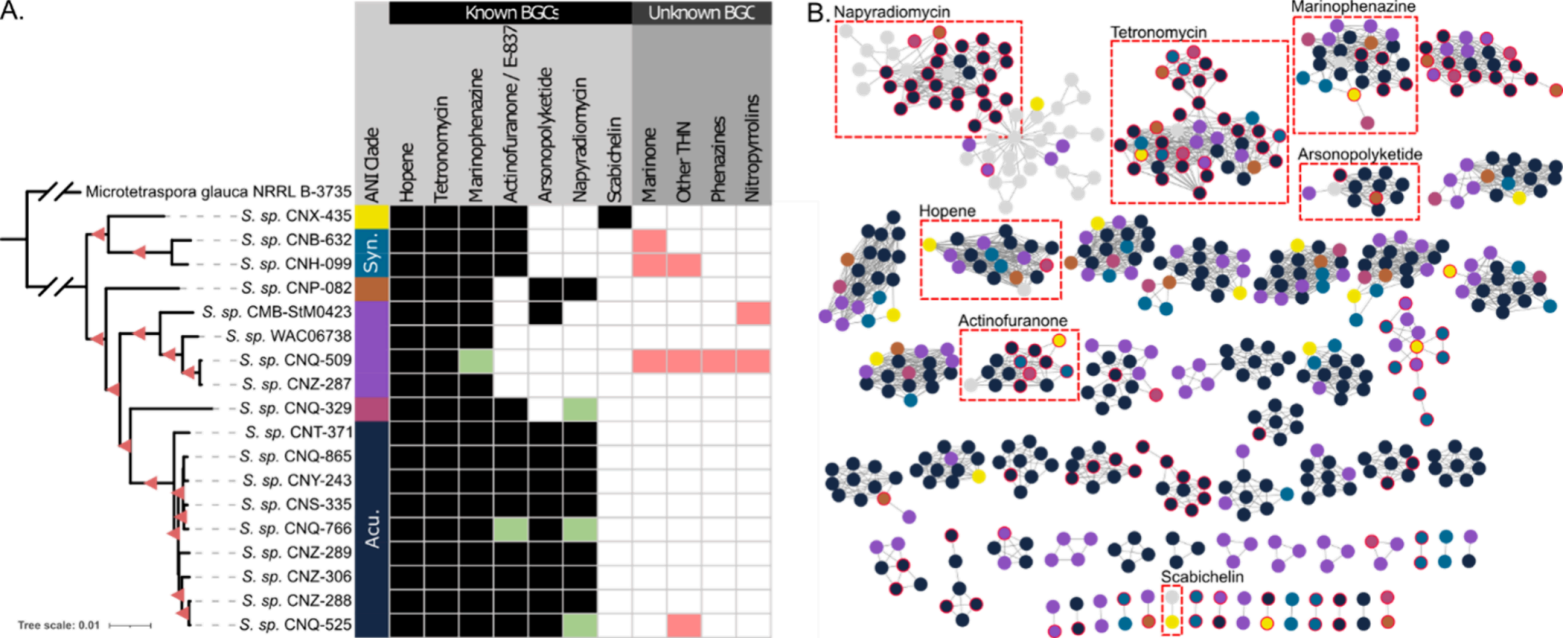
MAR4 multigene phylogeny and BGC distribution. (A) Multigene phylogeny delineates six <95% ANI clades (first column indicated by color). Clades correlated to *S. synnematoformans* (Syn.) and *S. aculeolatus* (Acu.) are indicated. The distributions of experimentally characterized BGCs (known) and those with predicted products (unknown) are presented as a presence/absence table. Black indicates the presence of the BGC, but compound production is not reported; green indicates both the presence of the BGC and compound production reported; red indicates the detection of compounds that have not been linked to a BGC. (B) BiG-SCAPE network of BGCs identified from MAR4 genomes. Nodes are colored by ANI species group; gray nodes are MIBiG reference BGCs. Nodes with red borders represent BGCs on contig edges. The seven MIBiG BGC families are boxed in red dashes. Singleton BGCs were omitted.

To understand how the biosynthetic capacity of the MAR4 genomes matched the reported chemistry, we used AntiSMASH^89^ and BiG-SCAPE^90^ to identify 608 BGCs (average 34 BGCs per strain) belonging to 173 gene cluster families (GCFs) based on homology, protein domain composition, and gene synteny. However, these numbers are likely inflated due to the fragmented assemblies that often split BGCs across multiple contigs. For instance, the napyradiomycin and tetronomycin BGCs were split across multiple GCFs. As such, the 18 GCFs that were related to experimentally characterized BGCs in the MIBiG reference database were reduced to seven after manual inspection. While higher quality assemblies are needed to quantify the full biosynthetic potential of the MAR4 clade, the vast majority of BGC and GCF diversity remains orphan in terms of the small molecules produced (Figure 4).

Of the characterized BGCs that could be confidently identified, those associated with tetronomycin (MIBiG #: BGC0000164), marinophenazine (MIBiG #: BGC0001221), and hopene (MIBiG #: BGC0001221) biosynthesis are conserved across all 18 MAR4 genomes, suggesting that these small molecules represent functional traits that help define the lineage. Notably, there were three highly similar GCFs that had strong similarities to the marinophenazine BGC. All MAR4 strains contain one of these GCFs, suggesting a common origin for all phenazines reported from the MAR4 clade. More work is needed to understand how highly similar BGCs can produce such a wide range of analogs as seen in the lavanducyanins, marinophenazines, and phenaziterpenes.

Given past observations of species-specific patterns in BGC distributions and compound production,^91,92^ we searched for patterns among the six ANI clades. In the context of a multigene phylogeny, each clade has a distinct BGC composition (Figure 4). Patterns are observed in the unnamed orange and purple clades, which include the only strains that lacked the actinofuranone BGC (MIBiG #: BGC0000050). Given its occurrence in all other MAR4 clades, it is likely that the actinofuranone BGC was present in the MAR4 common ancestor and subsequently was lost in these two clades. Similarly, the napyradiomycin BGC (MIBiG #s: BGC0001079 and BGC0000652) may have been present in a common ancestor of the *S*. *aculeolatus* and pink clades and possibly acquired by the orange clade via horizontal gene transfer (HGT). Interestingly, marinone production is only reported from strains that lack the napyradiomycin BGC, perhaps indicating functional replacement or extensive divergence from an ancestral THN BGC. Our analysis also shows that the arsonopolyketides BGC (MIBiG #: BGC0001283) is present in the *S. aculeolatus*, orange, and some members of the purple clade; however, no arsenic-containing metabolites have been reported from MAR4 strains. Finally, the scabichelin BGC (MIBiG #: BGC0000423) was only observed in the yellow clade, suggesting it was acquired by HGT. Additional genome sequencing will undoubtedly expand our understanding of the relationships between BGC distributions and MAR4 phylogeny.

## Conclusions

The MAR4 clade comprises a diverse group of predominantly marine-derived *Streptomyces* that account for at least six species based on ANI values, of which only two have been formally described. Strains within this clade have a remarkable capacity to produce halogenated hybrid isoprenoids, a phenotype supported by the relatively large numbers of PTase and VHPO genes observed in their genomes. Here, we compiled over 35 years of natural product research related to this group of bacteria. We identified 127 cultured MAR4 strains, an increase of 123% since the last assessment.^29^ Confounding their identification, we found as many as seven unique 16S rRNA gene sequences per MAR4 strain. While all copies fell within the larger MAR4 clade, they could be broadly distributed among the proposed ANI species, making species-level identification difficult using this gene.

While MAR4 strains are most commonly reported from marine samples, many of the terrestrial strains were isolated from agricultural rhizospheres where evapotranspiration associated with crop irrigation is known to increase soil salinity.^93^ Thus, adaptations that enhance survival in marine habitats could similarly facilitate growth in saline soils. While much remains to be learned about the ecology of MAR4 strains, their propensity to produce hybrid isoprenoids represents a defining feature of this lineage. Among the HI natural products reported, the *O*-prenylation observed in the marinophenazines is particularly rare, while the halogenation and prenylation patterns observed in the marinocyanins and lavanducyanins represent rare modifications to a phenazine core. The nitropyrrolins represent the first prenylated α-nitropyrroles to be discovered, and their biosynthesis remains unresolved. Similarly, the biosynthetic relationship of the marinones with the naphterpins, naphthablins, and naphthgeranines remains to be established. Unresolved questions also remain around the large diversity of napyradiomycins reported from different MAR4 strains, as they are difficult to account for given the conservation of the napyradiomycin BGC. While some may represent biosynthetic intermediates, the nonspecific oxidations observed on the terpene side chain and the loss of the unstable diazo group, which together account for much of the MAR4 napyradiomycin diversity, could be spontaneous.

Curiously, many MAR4 natural products have redox-active moieties such as quinones and phenazines, which can function as extracellular electron shuttles (EES) in both Gram-positive and Gram-negative bacteria.^94,95^ It is attractive to think that some of these compounds may perform a similar function in MAR4 strains. This creates an interesting paradox, as *Streptomyces* are obligate aerobes yet are known to survive in the absence of oxygen for extended periods of time.^96^ Evidence that some MAR4 natural products function as EESs^61^ suggests they may facilitate survival during periodic hypoxia events, which if established would add a new ecological role to the diverse compendium of compounds produced by these bacteria. Their ability to produce analogs could provide a selective advantage, as with phenazine production by *Pseudomonas aeruginosa*,^97^ where varying hydrophilicities are believed to facilitate biofilm distribution and support survival during low oxygen conditions.^95^

It is interesting to speculate that the enhanced production of HIs by MAR4 strains is an adaptation of the marine environment. Given that marine natural products are often more hydrophobic than their terrestrial counterparts,^98^ the increased lipophilicity introduced by the terpene moieties in MAR4 HIs could limit diffusion in seawater, thus allowing them to remain surface associated. While the ecological significance of enhanced HI production remains obscure, their discovery has fostered important advances in our understanding of natural product structures and their biosynthesis. Given the large number of orphan BGCs observed in MAR4 genomes, these bacteria will likely continue to yield unusual new natural products. In addition, the extant diversity of the MAR4 lineage remains unknown, and it is likely that additional species groups remain to be discovered. Resolving relationships among these groups, their BGC content, and the ecological functions of the compounds encoded in their genomes will continue to foster new discoveries and advance our understanding of how evolution drives natural product diversification.

## Experimental Section

### MAR4 Diversity

Gene sequences (16S rRNA) were collected using a text-based query of NCBI GenBank^99^ with the terms “MAR4”, “aculeolatus+16S”, and “synnematoformans+16S”. The resulting unique accession numbers were used to perform an iterative BLAST search against the nr/nt database using the “rentrez” R package and the top 5000 matches for each query compiled and duplicates removed. The resulting sequences were trimmed using the “GenomicRanges” and “seqinr” R packages, aligned on the MAFFT Web server (https://mafft.cbrc.jp/alignment/server/large.html) under default parameters, and a preliminary phylogeny generated with RAxML 8.2.12^100^ under the GTR + Gamma distribution model. Sequences that formed a monophyletic group with the initial query sequences were retained, becoming the new query sequences for the next iterative search (six in total). In addition, the 16S rRNA gene sequences from the *Streptomyces aculeolatus* and *S. synnematoformans* type strains were queried against the Joint Genome Institute Integrated Microbial Genomes (JGI-IMG) database,^101^ and the resulting sequences combined with those found above. A new phylogenetic tree was generated with these sequences using the methods described above, and MAR4 strains were identified as those within the least inclusive, monophyletic clade that contained the two type strains and those previously identified as “MAR4” in the literature. Distinct amplicon sequence variants (ASVs) were identified using VSEARCH.^102^

A broader *Streptomyces* phylogeny was generated using the *Streptomyces* Type Strain database available from the DSMZ List of Prokaryotic names with Standing in Nomenclature (https://lpsn.dsmz.de/genus/streptomyces/) and the MAR4 sequences identified above (Figure S1). MAR4 sequences shorter than 200 bp were removed to increase bootstrap support. The remaining sequences were aligned using the local SINA alignment package and the SILVA reference database “ref NR 99” with columns containing only gap characters removed. The ModelTest-NG application from the raxmlGUI^100,103^ package was used to select the GTR-GAMMA+I+G distribution model. RaxML 8.2.12 was used to generate a maximum likelihood tree by using the AutoMRE bootstrapping option. The tree was visualized using iTOL. Mothur 1.48.0^104^ was used to calculate pairwise percent similarities between MAR4 16S rRNA sequences with gap characters at the beginning and ends of the sequences ignored. A phylogeny was also generated after clustering the sequences at 98% identity using VSEARCH with sequences <1000 base pairs removed. The closest 10 LPSN strains from the previous tree were included in an ensemble alignment generated using MUSCLE,^105^ where the best-scoring alignment was extracted for tree building as described above. Corresponding metadata were determined through manual inspection of accession numbers.

### Genome Analyses

Results from the recursive BLAST searches included seven 16S rRNA sequences from MAR4 genomes. Text-based searches for known MAR4 strains reported in the literature yielded another 11 genomes. These genomes were used to generate a multilocus species phylogeny using the autoMLST^106^ Web server (https://automlst.ziemertlab.com/index) under default settings with *Microtetraspora glauca* NRRLB-3735 selected as the outgroup. Pairwise whole-genome ANI comparisons were performed using FastANI,^88^ and the output was visualized in R with “reshape2”, “ComplexHeatmap”, and “gplots”. BGCs were identified with AntiSMASH v6.0.^89^ Predicted BGCs were clustered into GCFs with BiG-SCAPE^90^ in mixed mode (family cutoff = 0.30, clan cutoff = 0.7) to identify similar BGCs among strains and GCF presence/absence data imported into Excel for manual analysis and visualization.

## Supporting Information Available

Table of strains identified by 16S rRNA as MAR4 *Streptomyces* and table of all natural products reported from MAR4 strains and reported bioactivities (XLSX)
Supplemental figures including 16S rRNA phylogeny and ANI analyses and structures of all natural products reported from MAR4 strains (PDF)
