# Supplementary material for: MAR4 Streptomyces: A Unique Resource for Natural Product Discovery
Source: J Nat Prod. 2024 Feb 14;87(2):439–52. doi: 10.1021/acs.jnatprod.3c01007 (PMC10897937; doi:10.1021/acs.jnatprod.3c01007)
Supplement: Supplementary file 2 — np3c01007_si_004.pdf [file np3c01007_si_004.pdf]

Supporting Information

MAR4 *Streptomyces:* A Unique Resource for Natural Product Discovery

Douglas Sweeney[1], Alexander B. Chase[2], Alexander Bogdanov[1], Paul R. Jensen[1]*

[1] Scripps Institution of Oceanography, University of California, San Diego, La Jolla, CA 92093, USA

[2] Department of Earth Sciences, Southern Methodist University, Dallas, TX 75275, USA

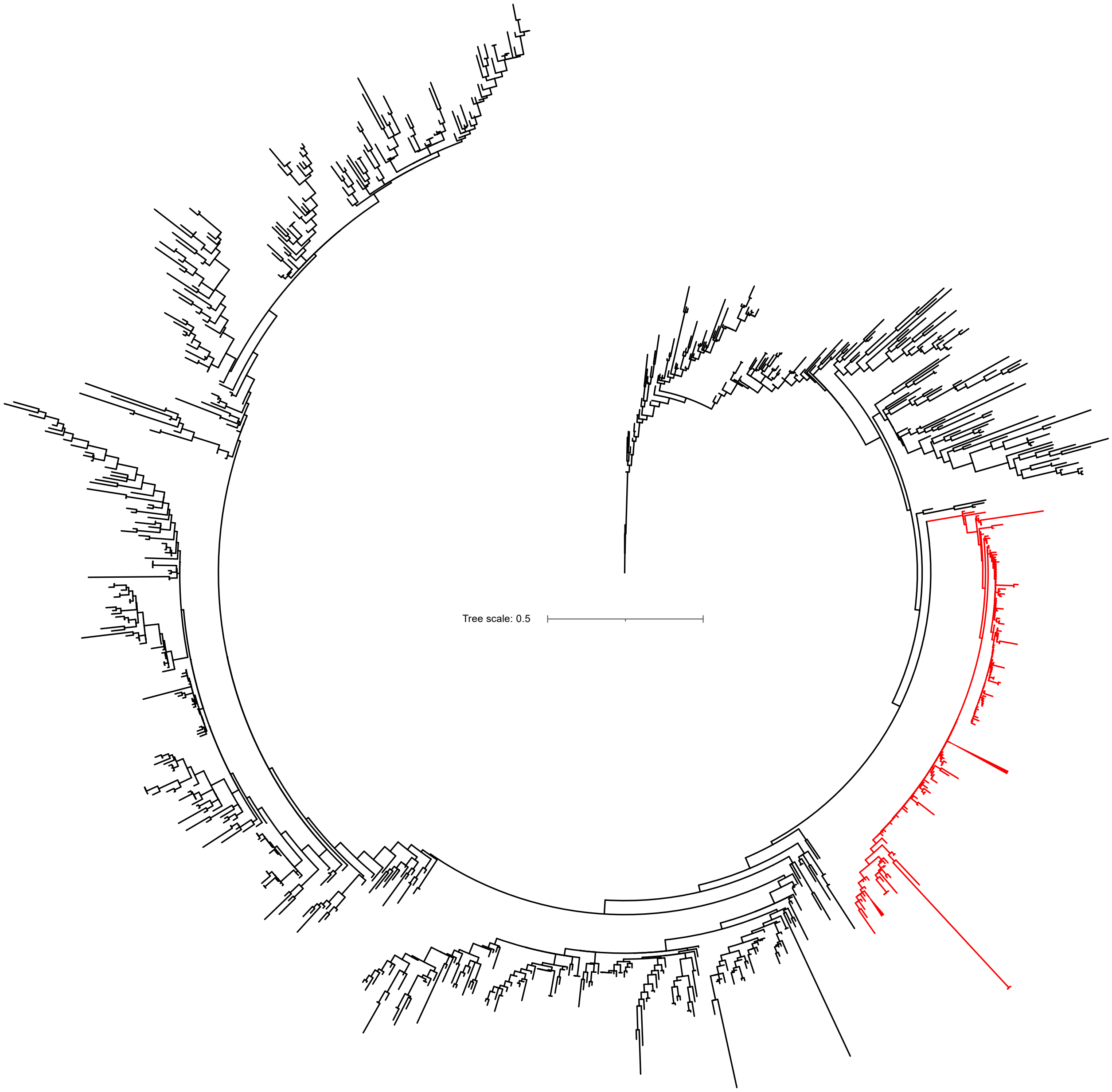

Figure S1. *Streptomyces* 16S rRNA gene phylogeny. Phylogeny includes all MAR4 16S rRNA gene sequences and all known *Streptomyces* type strains from the DSMZ LPSN. Red: MAR4 clade. See Figure 2 for detailed MAR4 phylogeny.

Chart S1. Molecules produced by MAR4 strains grouped into molecular families.

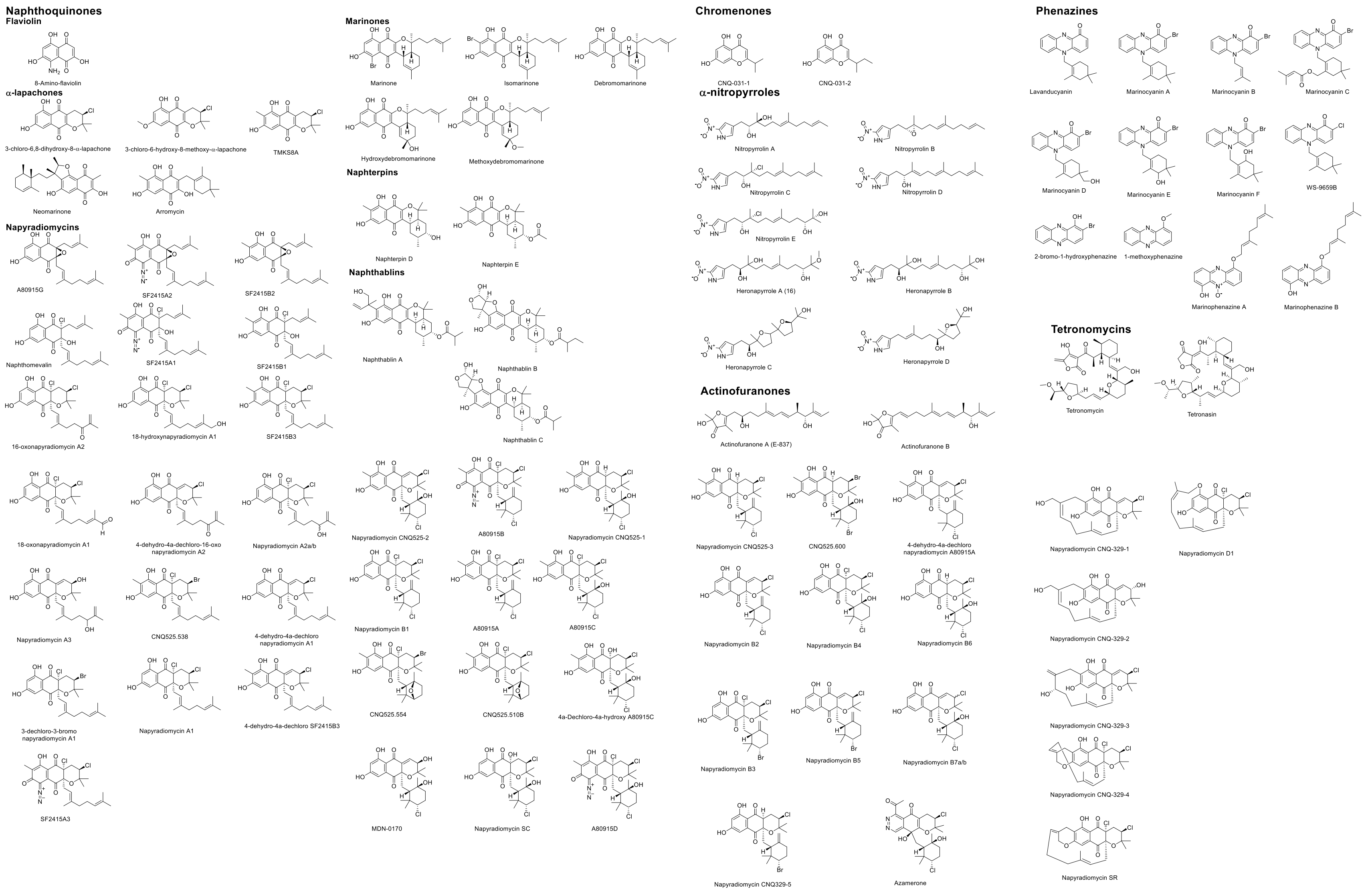

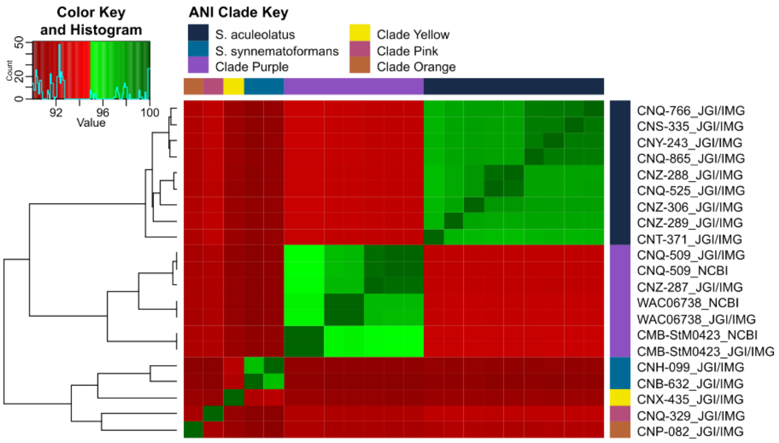

Figure S2. Pairwise ANI analysis of public genomes from NCBI GenBank and JGI/IMG. >95% ANI clades are indicated by colored bars. Genomes are labelled by strain number and database source. ANI clades associated with *S. aculeolatus* (dark blue) and *S. synnematoformans* (light blue) are indicated in the key along with four un-named clades, three of which are represented by only one genome.
